# Boys with Oppositional Defiant Disorder/Conduct Disorder Show Impaired Adaptation During Stress: An Executive Functioning Study

**DOI:** 10.1007/s10578-017-0749-5

**Published:** 2017-07-28

**Authors:** Jantiene Schoorl, Sophie van Rijn, Minet de Wied, Stephanie van Goozen, Hanna Swaab

**Affiliations:** 1 Department of Clinical Child and Adolescent Studies, Leiden University, P.O. Box 9555, 2300 RB Leiden, The Netherlands; 2 Leiden Institute for Brain and Cognition, P.O. Box 9600, 2300 RC Leiden, The Netherlands; 3 Department of Adolescent Development, Utrecht University, P.O. Box 80140, 3508 TC Utrecht, The Netherlands; 4 School of Psychology, Cardiff University, P.O. Box 901, CF10 3AT Cardiff, UK

**Keywords:** Stress, Executive functioning, Oppositional defiant disorder, Conduct disorder, Aggression

## Abstract

Evidence for problems in executive functioning (EF) in children with oppositional defiant disorder/conduct disorder (ODD/CD) is mixed and the impact stress may have on EF is understudied. Working memory, sustained attention, inhibition and cognitive flexibility of boys with ODD/CD (*n* = 65) and non-clinical controls (*n* = 32) were examined under typical and stressful test conditions. Boys with ODD/CD showed impaired working memory under typical testing conditions, and impairments in working memory and sustained attention under stressful conditions. In contrast to controls, performance on sustained attention, cognitive flexibility and inhibition was less influenced by stress in boys with ODD/CD. These results suggest that boys with ODD/CD show impairments in adaptation to the environment whereas typically developing boys show adaptive changes in EF.

## Introduction

Oppositional defiant disorder (ODD) and conduct disorder (CD) are developmental disorders [1], affecting around three percent of children, with somewhat higher rates in boys [2]. The Diagnostic and Statistical Manual of Mental Disorders (DSM-5) [1] defines ODD as a recurrent pattern of negativistic, defiant, disobedient, and hostile behavior towards authority figures. CD is characterized by a repetitive and persistent pattern of behavior in which the basic rights of others or major age-appropriate societal norms or rules are violated. Children with ODD/CD are at risk for a variety of negative outcomes: school dropout, unemployment, criminality and other psychiatric disorders such as depression and anxiety [3]. In order to be able to positively influence development and optimize outcome, it is important to understand the mechanisms driving aggressive behavior. One such underlying mechanism of behavior is executive functioning (EF). EF is crucial for controlling cognitive processing, emotions and behavior [4]. EF makes it possible to adapt behavior in situations that are new, complex, unpredictable, or have high load of information. Knowledge about EF in children with ODD/CD may help in understanding why they show antisocial and aggressive behavior, and thereby help in identifying targets for intervention.

There are several key EF functions: working memory, attention, inhibition, cognitive flexibility, planning and monitoring [4, 5]. This study focused on the first four EFs. Working memory involves holding information in mind and is for example necessary for considering alternatives, making action plans and reasoning. Attention involves being able to focus on what was chosen and suppress interference of other stimuli. Inhibition, or self-control, ensures control over one’s behavior, actions and emotions and requires withholding or delaying of responding. Cognitive flexibility means being able to adjust to changing demands or perspectives. Thus, EFs help us to focus on what’s necessary and regulate inappropriate behaviors, such as aggression.

There is an increasing acknowledgement of the role emotions play in EF. This is particularly interesting because in everyday life emotional or motivational influences are rarely absent [6]. Adequate functioning in emotionally charged or stressful situations requires flexibly adapting to changing environments. This may be compromised in children with ODD/CD, contributing to their antisocial and aggressive behavior. The importance of the impact emotions have on EF is also illustrated by research distinguishing between EF in neutral situations and EF in the context of affect, incentives and motivation, i.e., ‘cool’ and ‘hot’ EF [7]. Cool EF can turn into hot EF when a reward or punishment is introduced, thus when the situation becomes emotionally charged [8].

In ODD/CD samples EF deficits, more specifically cool EF, have been found, but to what extent they exist independently of ADHD comorbidity is controversial [9–11]. Some studies have found that EF impairments are more pronounced in those with ADHD and comorbid ODD/CD than ADHD alone [9]. Because comorbidity rates between ODD or CD and ADHD is high (59 and 43% respectively; [12, 13]) several studies have controlled for ADHD symptoms. However, results remain inconclusive; some find EF impairments after controlling for ADHD symptoms while others don’t. This is not surprising considering that EF deficits are associated with both conditions and covaried out when controlling for ADHD symptoms. Regarding type of EF impairment, studies that did find EF impairments reported various EF deficits: working memory, cognitive flexibility and planning impairments [14] or sustained attention and inhibition [15]. Dolan and Lennox [9], Fairchild et al. [16], Van Goozen et al. [17] and Woltering et al. [18], on the other hand, did not find cool EF impairments in adolescents with CD and children with ODD or externalizing behavior. Interestingly, all studies reported hot EF deficiencies [9, 14–18]. Evidence for impairments in hot EF in adolescents with ODD/CD is also found in studies examining decision making by using the Iowa Gambling Task [19]. Adolescents with high psychopathic traits [20] and adolescents with CD [21] were less likely to avoid risky choices than healthy adolescents.

So far studies have examined hot versus cool EF by comparing different paradigms. Studies using similar EF tests but using these in different, cool and hot, contexts are, to our knowledge, non-existent. Examining how negative emotions, as a result of a stressful condition (cool EF in a hot situation), affects EF is particularly important in children with ODD/CD. This knowledge may provide information about how their control over thought and behavior is modulated by stress. The aim of this study was to assess how EF is modulated by an established and ecologically valid psychosocial stressor. The stressor involved provocation, frustration and competition to increase emotional arousal in young children with ODD/CD. EFs of interest were working memory, sustained attention, inhibition and cognitive flexibility. In order to meet this aim, we examined EF under typical and under stressful conditions in boys with ODD/CD compared to non-clinical controls (NC). Previous literature found that those with ODD/CD show impairments in hot EF. Therefore, we hypothesized that boys with ODD/CD would perform worse than controls on EF tasks during stress.

EF dysfunctions have also been found in children diagnosed with ADHD or autism spectrum disorder (ASD) [11, 22], conditions which are often comorbid in children with ODD/CD [12, 23]. Considering that executive dysfunction may contribute to rigid behaviors, social difficulties, and difficulties in concentration and impulse control, this is not surprising. In order to assess whether EF deficits are not limited to those ODD/CD children with high levels of ADHD symptoms or autism traits, we also examined the relation of EF under typical and stressful conditions and ADHD symptoms and autism traits within the ODD/CD group. Although some studies found EF impairments independent of ADHD, it is thought that EF deficits in ODD/CD samples are only found when ADHD comorbidity has not been controlled for. We hypothesized that both ADHD symptoms and autism traits are inversely related to performance on the EF tasks under typical and stressful conditions.

## Method

The current study was approved by the Medical Ethical Committee of Leiden University Medical Centre (LUMC). Signed informed consent according to the declaration of Helsinki was obtained prior to participation.

### Participants

Participating boys visited Leiden University for 1 day with one of their parents. During this day parents completed the diagnostic interview schedule for children (DISC-IV) [24] and filled out questionnaires, while boys completed computer tasks, some of which were stress inducing. The second session took place approximately 2 weeks later either at the child’s school or the clinical health center where they were receiving clinical services. This order was fixed because a clinical assessment for inclusion of the study had to take place at the university first. The teacher of the child filled out the Teacher Report Form (TRF/6-18) [25].

*The ODD*/*CD group* was recruited at clinical health centers (*n* = 22), special education schools (*n* = 31) and regular elementary schools (*n* = 12). For the ODD/CD group (*n* = 65) inclusion criteria were a diagnosis of ODD or CD on the DISC-IV [24], an estimated IQ > 70 and age between 8 and 12 years old. They all met criteria for ODD diagnosis and 22 boys (34%) also met CD criteria. Other comorbid diagnoses were: ADHD (*n* = 45, 69%), anxiety (*n* = 38, 59%), depression (*n* = 9, 14%), and other disorders such as eating and tic disorders (*n* = 18, 28%), as based on the DISC-IV. Twenty boys had severe ASD traits (31%), indicated by score in the clinical range (T > 75) on the social responsiveness scale [26]. Twenty-five boys (38%) used psychostimulants and four (6%) used atypical antipsychotics.

*Non-clinical control group* All boys in the non-clinical control (NC) group (*n* = 32) were recruited at regular elementary schools. Inclusion criteria were an estimated IQ > 70, age between 8 and 12 years old, no ADHD diagnosis, low levels of autism traits expressed as a score in the normal range (T < 60) on the social responsiveness scale (SRS) [26]. and no severe aggressive behaviors, expressed as a diagnosis of ODD or CD, a score outside the normal range (T > 60) on the externalizing scale of the Child Behavior Checklist (CBCL/6-18) or TRF [25].

The ODD/CD group was similar in age (*M* = 10.3, *SD* = 1.28) and percentage of Caucasians (62%) compared to the NC Group (age *M* = 9.9, *SD* = 1.24), *t* = 1.37, *p* = .174; (Caucasian 66%), *χ* ^2^ = 0.15, *p* = .695. The ODD/CD group did have lower estimated IQ scores (*M* = 95.6, *SD* = 14.22) than the NC group (*M* = 104.8, *SD* = 12.10), *t* = −3.15, *p* = .002.

### Recruitment and Procedures

Boys referred through clinical centers were first screened with the CBCL [27]. Those who scored above the borderline cut off point on the externalizing scale were administered the DISC-IV interview Module E (section ODD and CD) [28]. Only those children who met criteria of either ODD or CD were asked to take part in this study. Special educational needs schools and regular elementary schools were selected based on their location, close to Leiden University with a maximum of approximately 1 h driving to Leiden University. Headmasters were contacted by one of the researchers and if the headmaster agreed to take part, information brochures for parents and response-cards were distributed by the teachers to the children in their class.

Participating boys were asked to visit Leiden University for 1 day with one of their parents. During this day parents signed an informed consent, filled out questionnaires and completed the DISC-IV interview. Boys completed the stress paradigm (see below) and filled out questionnaires. Within 2 weeks the second session took place either at the child’s school or at the clinical health center. The teacher of the child filled out the TRF [29] questionnaire afterwards.

#### Typical Versus Stressful Condition

The stressful condition was in a nonfamiliar laboratory at Leiden University, using an established and ecologically valid psychosocial stressor that involved provocation, frustration and competition to increase emotional arousal. Boys were led to believe that they were competing against a (videotaped) opponent of similar age and sex for the best performance and a favored award (for details, see [30, 31]). Three tasks were used to increase emotional arousal. Boys had to complete a simple reaction time computer task in which 16 of the 55 trials were randomly delayed by 6–12 s, causing frustration. The opponent gave negative feedback on their performance afterwards. Stress was further induced when boys had to play two competitive computer tasks and were led to believe they were playing against their opponent. First they played the ‘Door-opening task’ [32], in which boys had to open pre-programmed winning or losing doors. Opening a winning door meant receiving a coin, a losing door returning a coin. They were told that they could stop playing any time they wanted, but had to earn as much money as they could. Second, they played the ‘Hungry Donkey task’ [33] in which boys had to assist a hungry donkey by winning as many apples as possible by opening one of the four doors. Two doors resulted in gaining more apples than the other two doors, but the losses were also bigger, making them disadvantageous in the long run. After each task, the experimenters exchanged results via an intercom; according to protocol the opponent always had more money/apples, meaning the participant lost both tasks.

After having the boys provoked, frustrated and made them feel of losing out on winning the competition, the three EF tasks were performed in the following order: Sustained Attention Dots task, Spatial Temporal Span task and Shifting Attention Set task. To prevent learning effects, all EF tasks were practiced extensively attaining optimal performance before the actual performance started. There was no more communication with the opponent. Disclosure was done after completion of all EF tests; all boys were told they had won the competition after all and received their prize. None of the boys was aware at the time of testing that the video opponent was not a real participant.

Testing in the typical condition was done in a familiar environment, either at school or at the boy’s clinical health center, with no competition element, approximately 2 weeks after the stress condition. The three EF tasks were performed in the same order: Sustained Attention Dots task, Spatial Temporal Span task and Shifting Attention Set task. Testing was done according to typical neuropsychological testing protocols for children, with focus on positive support, re-assurance and efforts to have participants feel comfortable [34]. Similar to the stress condition, the EF tasks were practiced extensively before the actual task was performed, thereby preventing learning effects.

### Measures

*Estimated IQ* was measured with two subtests of the Wechsler Intelligence Scale for Children (WISC-III-NL) [35]: Vocabulary and Block Design. They have been found to provide a good estimation of full scale IQ scores [36].

#### EF

Working memory, sustained attention, inhibition and cognitive flexibility were measured using three subtests of the Amsterdam Neuropsychological Tasks (ANT) [37]. The ANT exists of 33 computer tasks and has been used in clinical and non-clinical populations (e.g., [38, 39]) and has satisfactory psychometric properties [40, 41]. Reliability and validity scores have been calculated for subsets of tasks, including those used in this study. Test–retest reliability is moderate to high, with coefficients ranging between 0.70 and 0.85 for reaction times (RTs) in different tasks [42–44]. Various studies provide evidence for construct validity and discriminant validity of the ANT [44–48]. Children were given a verbal instruction and practiced each task before the actual task started. As a result their performance was most optimal and learning effects were prevented the second time the task was performed.

*Working memory* was measured by the Spatial Temporal Span task [37]. This task consists of nine squares, presented in a three-by-three square. During each trial a sequence of these squares was pointed at, and boys had to reproduce the sequence in backwards order. The parameter used in this study was the number of correctly identified squares in the correct backward order. Expected score ranges are between 4 and 162, with higher scores indicating better working memory.

*Sustained attention* was measured with the Sustained Attention Dots task [37], a continuous performance task. 600 dot patterns with 3, 4 or 5 dots are presented to the boys. Boys had to press the mouse button with their dominant hand if a 4 dot pattern was shown on the screen. If a 3 or 5 dot pattern was shown they had to press the mouse button with non-dominant hand. The parameter used in this study is percentage of errors (misses), controlled for average time to completion (response time).

*Inhibition* was measured with the Shifting Attention Set task [37]. This task consists of a horizontal bar containing ten squares. In part 1 a green square moves across the bar in a randomly varied direction. Boys had to give a compatible response. They had to follow the green square (movement to the left means pressing the left mouse button and vice versa). Part 2 requires an incompatible response. Now a red square moves across the bar in a randomly varied direction and boys had to press the mouse button in opposite direction (if the square moves to the right press the left mouse button). Inhibition was calculated by subtracting Part 1 from Part 2. Parameters are average time to completion (response time) controlled for the number of errors.

*Cognitive flexibility* was also measured with the Shifting Attention Set task [37]. In Part 3 of this task green and red squares are randomly shown in varied directions. Boys had to combine the instructions of Part 1 and Part 2. If the square is green they had to give a compatible response (same-side). If the square is red they had to give an incompatible response (opposite side). Switching between these unpredictable competing response requires cognitive flexibility. The difference in time to completion (response time) and number of errors of the compatible responses of Part 1 were subtracted from Part 3.

#### Mood Report

As part of the stress condition, the manipulation of psychological state was checked with an adapted version of the Von Zerssen’s clinical self-rating scale [49] containing 11 moods (happy, well, cheerful, good, liked, satisfied, afraid, worried, embarrassed, ashamed, angry) and feeling of control, which boys rated on a five-point scale ranging from positive towards negative feelings (e.g. 1 = happy, 5 = gloomy) (see also [31]. All moods were combined into one negative mood score. Mean Cronbach’s alpha was 0.86.

We also asked boys to rate on a five-point scale who they thought would win the competition (they or the opponent). We used three ratings in time, one before the competition started, one before the EF tasks were administered and one after completion of the EF tasks, but before the competition outcome.

### Statistical Analysis

Some data was missing due to equipment dysfunction or discontinuation of participation in the study. Based on this, sample sizes varied from 97 to 89 boys per analysis. To assess the effect of stress on EF (working memory, sustained attention, inhibition and cognitive flexibility) repeated measures ANOVA’s (RANOVA’s) were performed with group (ODD/CD vs. NC) as between subjects factor and condition (typical vs. stress) as within subject factor. Next, within the ODD/CD group a correlation analysis was performed to examine the possible relation between EF under typical and stressful conditions and ADHD symptoms and autism traits. Eta squared (η^2^) effect sizes were calculated with 0.02 being a small, 0.13 a medium and 0.26 a large effect [50].

## Results

Because IQ was significantly higher in the NC group than the ODD/CD group a correlation analysis was performed between IQ and the various EF measures. IQ was significantly related to working memory in stress (*r* = .22, *p* = .032) and typical conditions (*r* = .28, *p* = .007), sustained attention under typical conditions (*r* = −.25, *p* = .018), inhibition under typical conditions (*r* = −.23, *p* = .027) and cognitive flexibility under stress (*r* = −.42, *p* < .001) and typical conditions (*r* = −.23, *p* = .027). In the following RANOVA’s IQ was therefore included as a covariate.

A MANOVA revealed that medication use was not related to the EF measures, *F* (14, 41) = 1,21, *p* = .304. Therefore, medication use was not controlled for in subsequent analyses.

### Stress Manipulation

First, the effect of the stress manipulation was checked by analyzing mood change due to the psychosocial stressor. There was a significant main effect of stress, *F* (1, 92) = 38.13, *p* < .001, with a large effect, η^2^ = 0.29, but no effect of group *F* (1, 92) = 0.04, *p* = .835, nor was there a stress by group interaction *F* (1, 92) = 0.72, *p* = .399. Both the ODD/CD and NC group reported more negative mood when stress was induced (ODD/CD *M* = 1.7 *SD* = 0.59, NC *M* = 1.8 *SD* = 0.57 versus ODD/CD *M* = 2.5 *SD* = 1.05, NC *M* = 2.4 *SD* = 0.92), indicating that stress induction was successful and equal in both groups.

Both groups reported at the beginning of the competition that they thought they would win, which changed during the competition towards the believe that the opponent would win, *F* (1, 89) = 53.86, *p* < .001, with a large effect η^2^ = 0.38. There were no group differences *F* (1, 89) = 0.01, *p* = .911, and there was no stress by group interaction, *F* (1, 89) = 0.20, *p* = .653.

After the last EF task, but before the competition outcome, both groups still reported more negative mood than before the competition *F* (1, 91) = 14.93, *p* < .001, with a medium effect η^2^ = 0.14, and were still less confident about winning the competition *F* (1, 89) = 10.67, *p* = .002, η^2^ = 0.11; however, there was again no main effect of group *F* (1, 91) = 0.00, *p* = .976, *F* (1, 89) = 0.09, *p* = .767, or stress by group interaction, *F* (1, 91) = 0.34, *p* = .562, *F* (1, 89) = 0.02, *p* = .895.

### Working Memory

There was a significant main effect of group, *F* (1, 91) = 8.45, *p* = .005, η^2^ = 0.09, with the ODD/CD group generally performing worse, no effect of condition, *F* (1, 91) = 0.33, *p* = .565, and no condition by group interaction, *F* (1, 91) = 0.32, *p* = .571 (see Fig. 1.1). The covariate IQ did not have a significant effect, *F* (1, 91) = 0.60, *p* = .441 (see Table 1 for *M* and *SD* scores). Post hoc ANCOVA’s revealed that the ODD/CD group performed worse under typical conditions, *F* (1, 95) = 6.20, *p* = .015, η^2^ = 0.06, and stress conditions, *F* (1, 96) = 4.47, *p* = .037, η^2^ = 0.05, than controls.

### Sustained Attention

There was a significant main effect of group, *F* (1, 86) = 4.79, *p* = .031, η^2^ = 0.05, and condition by group interaction, *F* (1, 86) = 6.47, *p* = .013, η^2^ = 0.07, but no effect of condition, *F* (1, 86) = 0.42, *p* = .520 (see Fig. 1.2 and Table 1). There was an effect of the covariate IQ, *F* (1, 86) = 4.77, *p* = .032 η^2^ = 0.05, response time stress condition, *F* (1, 86) = 39.62, *p* < .001 η^2^ = 0.23, and response time typical condition, *F* (1, 86) = 6.47, *p* = .013 η^2^ = 0.07.

Post hoc paired sample *t*-tests revealed that performance of the ODD/CD group did not differ between stress and typical conditions, *t* = 0.25, *p* = .805, whilst for the controls it did, *t* = 3.84, *p* = .001. Post hoc ANCOVA’s revealed that the ODD/CD group only performed worse than the NC group under stressful conditions, *F* (3, 97) = 8.49, *p* < .001, η^2^ = 0.22, but not under typical conditions, *F* (3, 91) = 0.17, *p* = .683.

### Inhibition

There was a significant condition by group interaction, *F* (1, 86) = 4.75, *p* = .032, η^2^ = 0.05, but no significant effect of condition *F* (1, 86) = 0.03, *p* = .863 or group *F* (1, 86) = 1.14, *p* = .289 (see Fig. 1.3). This finding was specific for response time (RT), as no significant effects were found when analyzing errors (rather than RT) in an additional RANOVA. The covariates IQ *F* (1, 86) = 0.75, *p* = .390 and number of errors stress condition *F* (1, 86) = 0.97, *p* = .327 did not have a significant effect, whereas the number of errors typical condition did, *F* (1, 86) = 7.79, *p* = .006. For mean and *SD* scores see Table 1.

Post hoc paired sample *t*-tests revealed that the performance of the ODD/CD group was not affected by varying testing conditions, *t* = -1.51, *p* = .136, whereas the NC group responded slower (RT increased) during the stressful condition, *t* = -3.88, *p* = .001. Post hoc ANCOVA’s revealed that performance of both groups did not differ from each other under typical conditions, *F* (2, 89) = 0.00, *p* = .993, or under stressful conditions, *F* (3, 94) = 2.15, *p* = .146.

### Cognitive Flexibility

There was a significant condition by group interaction *F* (1, 86) = 6.62, *p* = .012, η^2^ = 0.07, but no effect of condition *F* (1, 86) = 2.10, *p* = .151 or group *F* (1, 86) = 1.23, *p* = .271 (see Fig. 1.4). Again, this finding was specific for RT, there were no significant effects when analyzing errors (rather than RT) in an additional RANOVA. The covariates IQ *F* (1, 86) = 0.69, *p* = .409, number of errors stress condition *F* (1, 86) = 0.49, *p* = .487 and number of errors typical condition, *F* (1, 86) = 0.17, *p* = .682, did not have a significant effect. For mean and *SD* scores see Table 1.

Although post hoc paired samples *t*-tests showed that both the ODD/CD group, *t* = −2.44, *p* = .018, and the NC group, *t* = −4.95, *p* < .001, responded slower during the stressful condition, the interaction effect of the RANOVA indicates that the NC group adapted more. Post hoc ANCOVA’s revealed that the ODD/CD group responded faster than the NC group under stressful conditions, *F* (3, 95) = 4.45, *p* = .038, η^2^ = 0.05, but no differences were found under typical conditions, *F* (3, 93) = 0.17, *p* = .680.

**Fig. 1:**
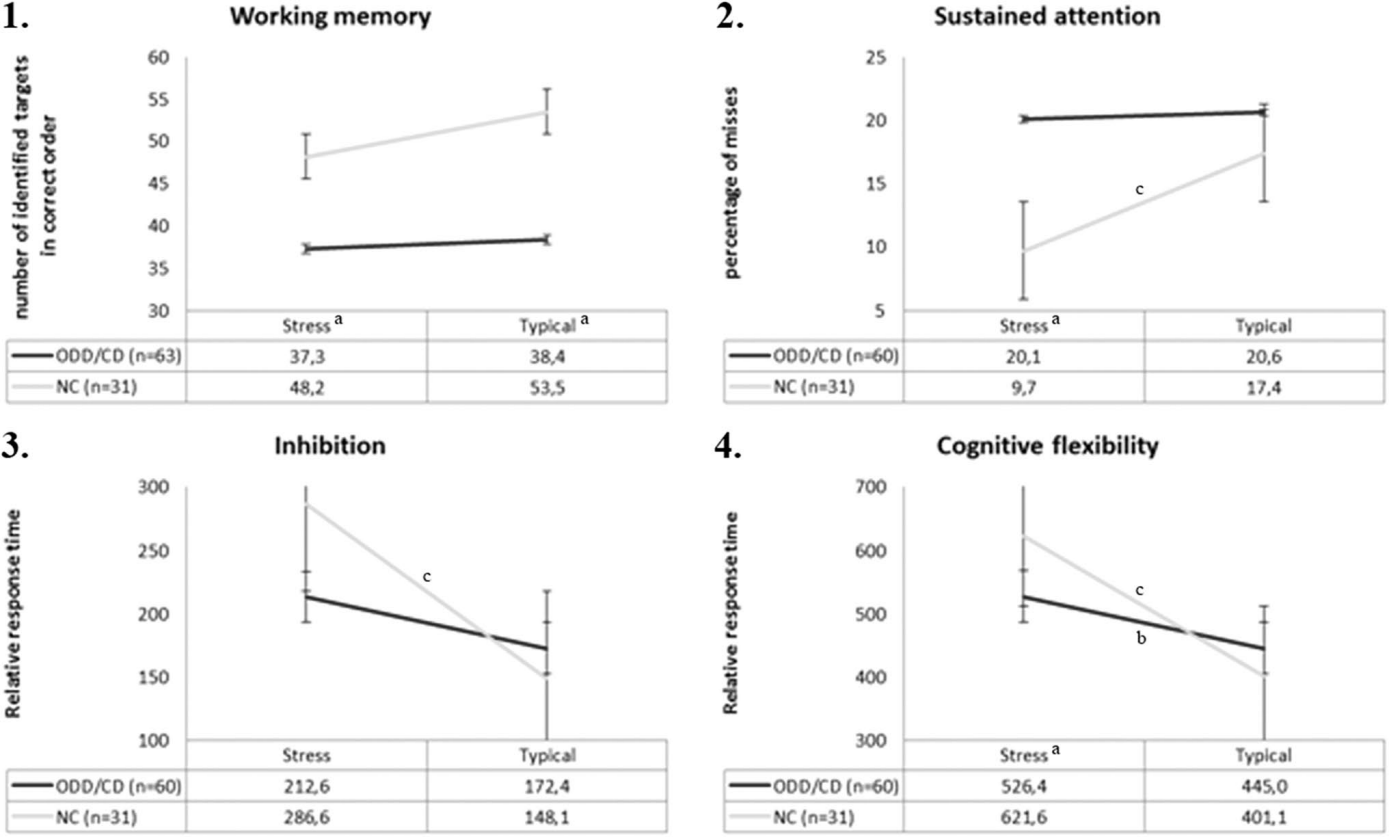
Increase and decrease of EFs during typical and stressful test conditions in the ODD/CD and NC group. Significant interaction effects were found for sustained attention, inhibition and cognitive flexibility. *a* Significant group difference between ODD/CD and NC group. *b* Significant difference between stressful and typical test conditions in the ODD/CD group. *c* Significant difference between stressful and typical testing conditions in the NC group

**Table 1:** Means, SDs in the ODD/CD and NC groups for EF in typical and stressful test conditions

		ODD/CD	NC	Group by condition effect	Within group effect	Within condition effect
Working memory	Stress	37.3 ± 17.65	48.2 ± 18.94	*F (*1, 91) = 0.32 *p* = .571	*F (*1, 91) = 8.45 ***p*** = .**005**	*F (*1, 91) = 0.33 *p* = .565
	Typical	38.4 ± 20.86	53.5 ± 19.90			
Sustained attention	Stress	20.1 ± 15.40	9.7 ± 4.27	*F (*1, 86) = 6.47 ***p*** = .**013**	*F (*1, 86) = 4.79 ***p*** = .**031**	*F (*1, 86) = 0.42 *p* = .520
	Typical	20.6 ± 11.84	17.4 ± 12.40			
Inhibition	Stress	212.6 ± 196.22	286.6 ± 199.17	*F (*1, 86) = 4.75 ***p*** = .**032**	*F (*1, 86) = 1.14 *p* = .289	*F (*1, 86) = 0.03 *p* = .863
	Typical	172.4 ± 202.27	148.1 ± 184.62			
Cognitive flexibility	Stress	526.4 ± 268.51	621.6 ± 302.54	*F (*1, 86) = 6.62 ***p*** = .**012**	*F (*1, 86) = 1.23 *p* = .271	*F (*1, 86) = 2.10 *p* = .151
	Typical	445.0 ± 199.27	401.1 ± 185.12			

Significant effects are in bold

### EF During Stressful and Typical Conditions in Relation to ADHD Symptoms and Autism Traits

The correlation analysis revealed that EF during stressful and typical conditions were not related to ADHD symptoms or autism traits in boys with ODD/CD (see Table 2).

**Table 2:** Statistics of the correlation analysis of EF under typical and stressful test conditions and ADHD symptoms and autism traits within the ODD/CD group

	ADHD symptoms		Autism traits	
	*r*	*p*	*r*	*p*
Stress				
Working memory	−0.07	0.578	0.04	0.768
Attention	−0.04	0.779	0.05	0.684
Inhibition	0.02	0.907	−0.11	0.417
Cognitive flexibility	0.07	0.583	−0.8	0.518
Typical				
Working memory	−0.15	0.250	0.06	0.622
Attention	0.03	0.798	0.05	0.689
Inhibition	−0.09	0.473	−0.22	0.093
Cognitive flexibility	0.00	0.971	−0.06	0.670

## Discussion

The aim of this study was to assess whether boys with ODD/CD have EF impairments, and whether EF is modulated by stress, in boys with ODD/CD, focusing on a broad range of EF domains. These domains were working memory, sustained attention, inhibition and cognitive flexibility. In order to meet this aim, we examined how an established and ecologically valid psychosocial stressor that involved provocation, frustration and competition to increase emotional arousal, effects EF in boys with ODD/CD compared to NC.

The main finding of this study is that in stressful situations, deficits in adaptation to the environment in boys with ODD/CD became more prominent; whereas typically developing boys showed adaptive changes in EF, this was largely lacking in boys with ODD/CD.

The ODD/CD group showed impairments in working memory in typical conditions. This finding fits with studies from Syngelaki et al. [14] who reported working memory impairments in young offenders and Seguin i.e., [51, 52] who stated that physical aggression and the violent behavior symptoms of CD are related to working memory deficits; though in two child populations with ODD or externalizing behavior this was not found [17, 18]. When stress was added, boys with ODD/CD still showed EF impairments in the domain of working memory. In addition, they now also showed impairments in sustained attention. Thus, under stress, boys with ODD/CD had more impaired EF functioning than typically developing boys. This fits with other studies on hot EF, showing that children with ODD/CD have hot EF impairments [9, 14–16, 20, 21].

Crucial to our aim of examining how stress impacts EF, the interaction effects showed us that whereas performance in controls in specific domains of EF (sustained attention, inhibition, cognitive flexibility) changed as a result of increasing stress, performance of boys with ODD/CD was less influenced in these domains. Although there was some change in cognitive flexibility, sustained attention and inhibition were not at all influenced by stress in boys with ODD/CD. Self-reports revealed that negative mood was equally induced in both groups, yet their performance in EF tasks differed. These negative emotions may have led to an adaptation in performance of controls, whereas for boys with ODD/CD these negative emotions only influenced their performance in cognitive flexibility. Although controls showed longer response times for inhibition and cognitive flexibility in the stressful condition, they did not make more errors. This indicates that they adapted their speed of responding so that they had sufficient time for accurate responses, which is an adaptive response. In times of stress the body responds with shifts in interactions within and between brain networks to optimize responses [53]. For example, during a physical attack cognitive processes to identify the location of the attacker become very important and are giving priority. In our study the control group responded (automatically) to the environmental contingencies. Thus adding stress resulted in more adaptive responses in the control group, but this was absent in the ODD/CD group for sustained attention and inhibition and to a lesser extend for cognitive flexibility. This finding indicates that boys with ODD/CD may have difficulties in adapting their behavior to an optimal level in emotional, demanding environments.

This finding that boys with ODD/CD did not change their performance much can be explained by Yerkes and Dodson’s law [54]. An inverted U shape describes the relation between arousal and performance. An optimal level of arousal increases performance while too much or too little impairs performance. Boys with ODD/CD may have different arousal levels than typically developing children. As a result they may not be able to benefit from increased arousal to the same degree as typically developing children. Although in this article we considered boys with ODD/CD as one group, in a previous study [30] on variability in arousal levels in boys with ODD/CD, the same stressor led to some being overaroused and some being underaroused with distinct relations to behavioral problems. According to the Yerkes–Dodson law both are unfavorable for optimal performance. Another possibility is that boys with ODD/CD are less able to use their physiological signs to adjust their behavior. Furthermore, they might not have other strategies to deal with changing demands. Yet another possibility is that only in the control group negative mood motivated them to increase their performance [55]. Unfortunately, we did not control for motivational aspects. We asked all boys to rate who they thought would win the competition. But we do not know if the motivation to win the competition was similar in both groups. To find out why boys with ODD/CD did not respond similar to controls needs to be studied in more detail.

One of the main findings of this study is that inducing stress made differences in adaptation visible between boys with ODD/CD and controls. Adding stress had a different effect on sustained attention, inhibition and cognitive flexibility in boys with ODD/CD than controls. This implicates that in complex or emotional situations boys with ODD/CD may experience difficulties in adaptation. Failure to flexibly adapt in complex or changing environments is important for adequate functioning in daily life, and thus may contribute to behavioral problems of those with ODD/CD. This idea is supported by another study in which it was found that boys with ODD/CD made less economic and less adaptive decisions when the situation was ambiguous and emotionally charged [56]. Emotions are important in guiding behavior adaptively to the environment [57]. One of the mechanisms driving aggressive and antisocial behavior may therefore be the inability to use emotions in behavioral adaptation. Our findings may have implications for clinical diagnosis and treatment of children with ODD/CD. Although boys with ODD/CD reported increased negative mood this did not result in behavioral adaptation. Therefore, it might help if boys with ODD/CD learn how to use their emotions to adapt their behavior. For example, they might need help in identifying their emotions and how to regulate these emotions in acceptable way. The specific deficit in their working memory abilities could hamper thinking about strategies. Working memory is involved in thinking about alternatives while holding information in mind and problem solving. Practicing different emotion regulation strategies might help them to respond in a socially acceptable way in emotional situations because it will take less of an effort to think of alternative behavior. Thus, the emotional context of behavior should be taken into account and be a target of support and intervention. Also, future studies examining EF should carefully consider under what circumstance they examine EF. Recently, a study who measured Stroop interference (measuring selective attention and cognitive flexibility) under distressing and neutral emotional stimulation, showed that adolescent males with CD had impaired cognitive control when exposed to distressing emotional stimuli compared to controls, thus when cognitive demand is high, but not when exposed to neutral stimuli [58]. Woltering, et al. [18] also demonstrated the value of adding emotions to typical cool EF tasks. Their adapted Go-Nogo task proved that only when emotion was induced children with externalizing behavior performed worse than controls; in the original cool EF Go-Nogo task no differences were found.

EF dysfunction has often also been found in children diagnosed with ADHD or ASD [11, 22], conditions which are found comorbid in children with ODD/CD [12, 23]. Considering that executive dysfunctioning may contribute to rigid behaviors, social difficulties, and difficulties in concentration and impulse control, this is not surprising. Therefore, we also examined if ADHD symptoms and autism traits were related to EF within the ODD/CD group. None of the EFs under typical and stressful conditions were related to ADHD symptoms and autism traits in boys with ODD/CD. So the EF impairments existed in the ODD/CD group independent of their level of ADHD symptoms and autism traits.

A limitation of this study is that we included only boys. Although problems with aggressive and antisocial behavior are found in girls as well e.g., [59], the boy to girl ratio is skewed. Larger samples are needed to assess potential gender differences in the phenotype and underlying neurodevelopmental mechanisms.

Also, studies on non-clinical children and adolescents indicate that EF, especially hot EF, is still under development into adulthood [60, 61]. It would have been interesting if we had expanded our age range to late adolescence, this may have provided us further insights into the impact of stress on EF in relation to ODD/CD. Our design did not allow for counterbalancing the stressful and typical test condition. However, learning effects did not occur since performance was sometimes better in controls (sustained attention) during the first time (i.e., stressful condition) the task was administered.

Taken together we found evidence of EF deficits in boys with ODD/CD and importantly we found that while controls adapted their behavior in demanding environments, boys with ODD/CD did so only for cognitive flexibility. Failure to adapt behavior may underlie some of the maladaptive behaviors of boys with ODD/CD in complex and emotionally charged situations, situations that are especially vulnerable to elicit aggressive and antisocial behavior.

## Summary

The present study examined whether boys with ODD/CD (*n* = 65) showed difficulties in EF compared to non-clinical control boys (*n* = 32) and whether stress may have an impact on EF in these two groups. EFs that were studied were working memory, sustained attention, inhibition and cognitive flexibility. All boys were assessed under typical test conditions and under stressful test conditions. The stress condition existed of an established and ecologically valid psychosocial stressor that involved provocation, frustration and competition to increase emotional arousal. Results showed that boys with ODD/CD showed impaired working memory under typical testing conditions, and impairments in working memory and sustained attention under stressful conditions. In contrast to controls, performance on sustained attention, cognitive flexibility and inhibition was less influenced by stress in boys with ODD/CD. These results suggest that boys with ODD/CD show impairments in adaptation to the environment whereas typically developing boys show adaptive changes in EF. The failure of boys with ODD/CD to flexibly adapt their behavior may underlie some of their aggressive and antisocial behavior in complex and emotionally charged situations.
